# Supplementary figures and images for: Benefits of dance for Parkinson’s: The music, the moves, and the company
Source: PLoS One. 2022 Nov 21;17(11):e0265921. doi: 10.1371/journal.pone.0265921 (PMC9678293; doi:10.1371/journal.pone.0265921)

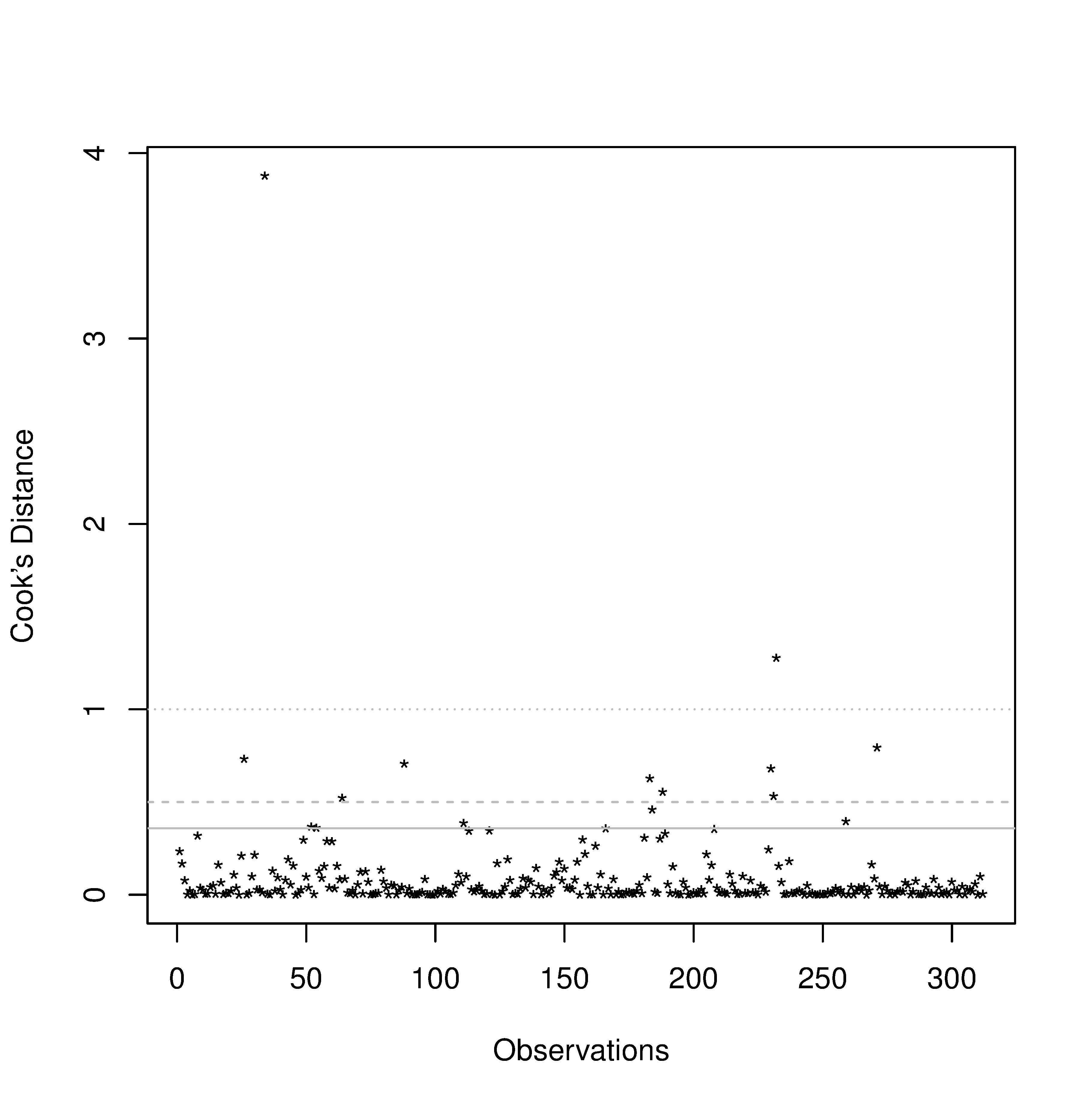

Supplement: S1 Fig — Outliers identified by Cook’s Distance. Grey line represents the cut-off point at 0.358, corresponding to 4 times the mean. Dotted lines at 1 and 0.5 Coo’s Di. X-axis = Observations, y-axis = Cook’s distance of the null-model’s residuals. (TIF) [file pone.0265921.s001.tif]

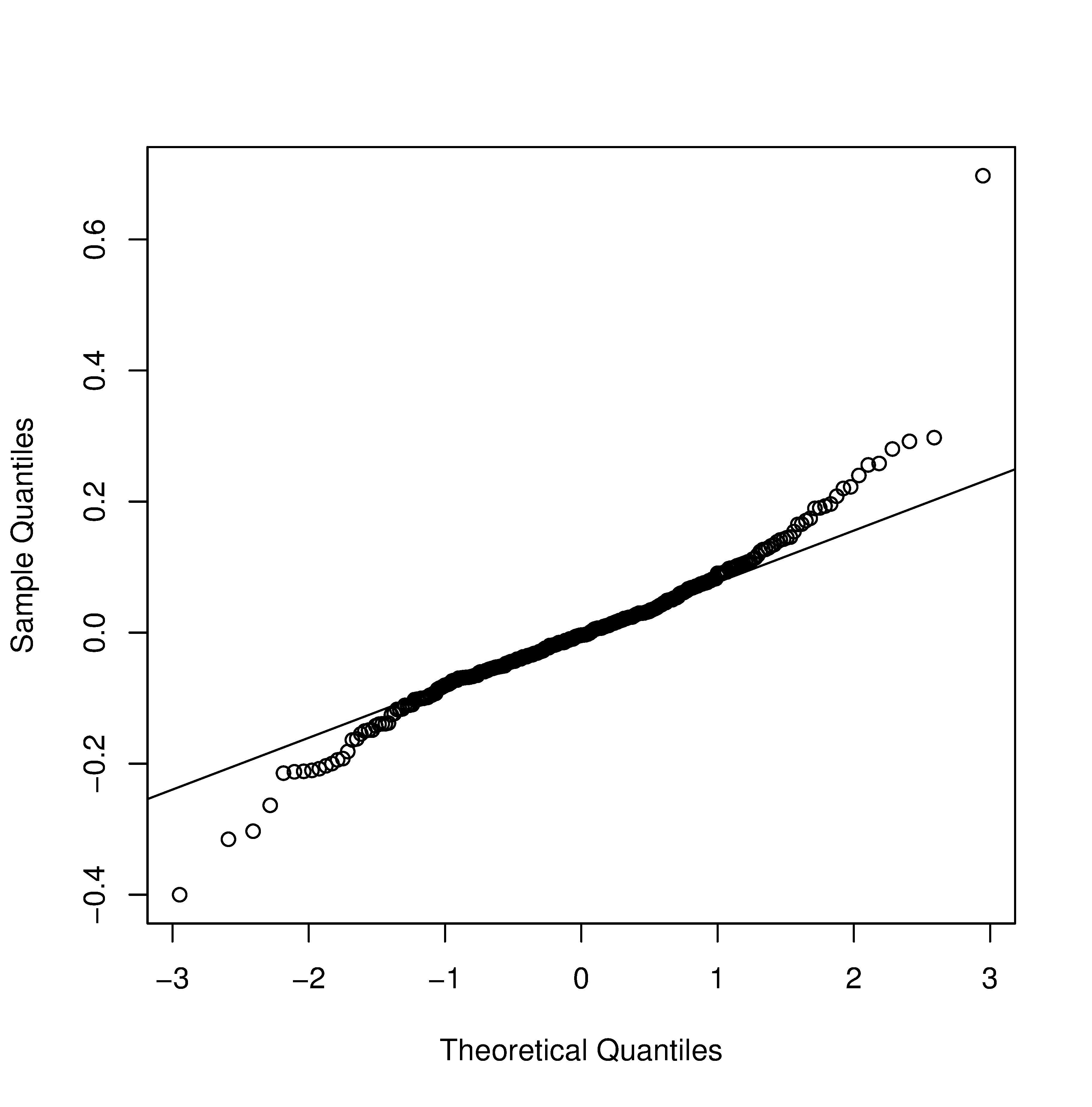

Supplement: S2 Fig — Residuals of null-model before outlier correction. (TIF) [file pone.0265921.s002.tif]

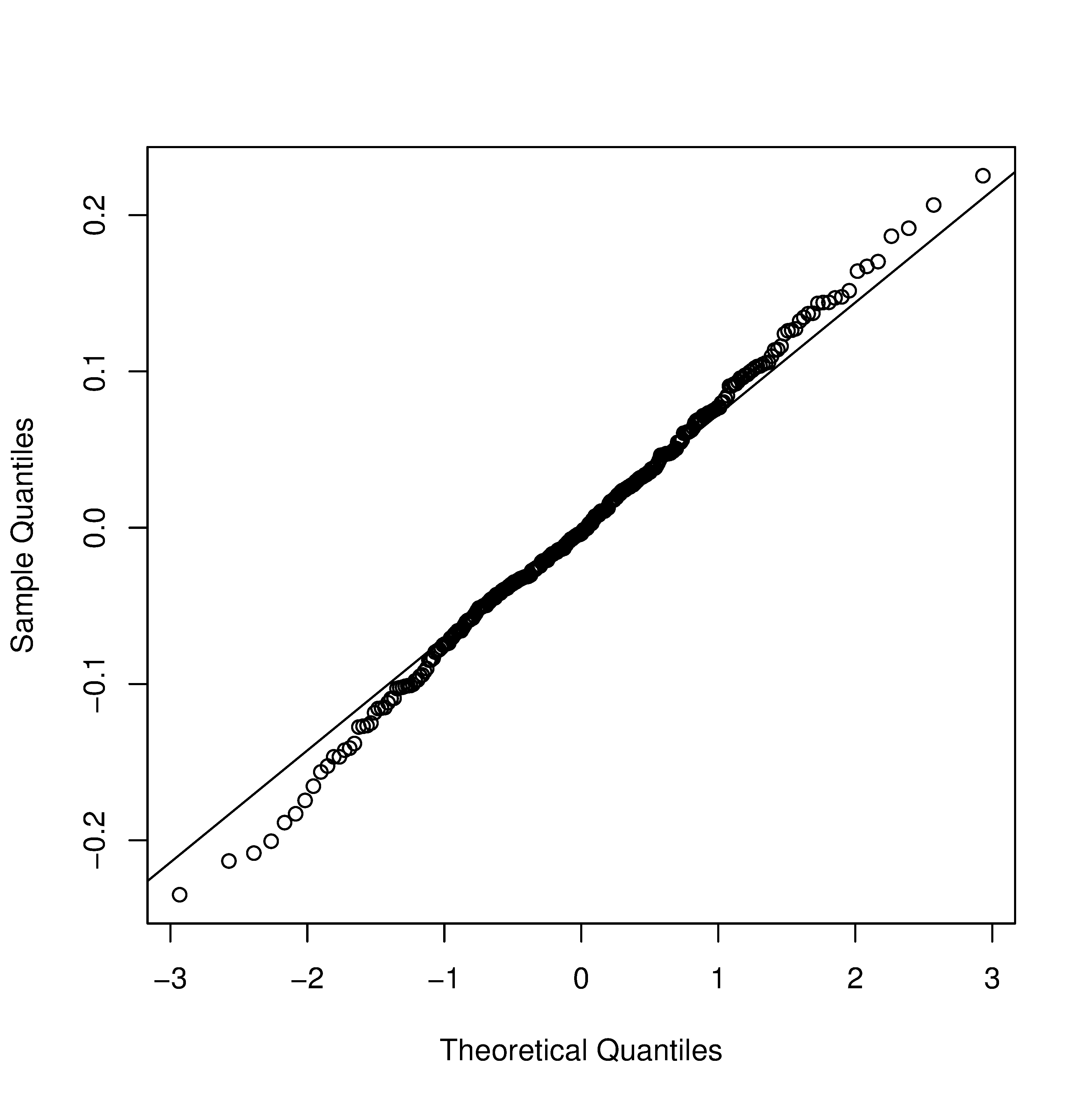

Supplement: S3 Fig — Residuals of null-model after outlier correction. (TIF) [file pone.0265921.s003.tif]
