# Supplementary material for: Benefits of dance for Parkinson’s: The music, the moves, and the company
Source: PLoS One. 2022 Nov 21;17(11):e0265921. doi: 10.1371/journal.pone.0265921 (PMC9678293; doi:10.1371/journal.pone.0265921)
Supplement: S1 Table — Descriptive data reported for Mean Timed Up and Go (TUG) test in seconds and SD for standard deviation for each condition based on the original data without the outlier trials. (DOCX) [file pone.0265921.s004.docx]

| **Music** | **Time** | **Mean [s]** | **SD** |
| --- | --- | --- | --- |
|  |  |  |  |
| Without Music | Before dance | 12.53 | 3.87 |
| With Music | Before dance | 11.91 | 3.10 |
| Without Music | After dance | 11.83 | 3.31 |
| With Music | After dance | 11.86 | 3.37 |

S4 Table. Descriptive Statistics. Descriptive data reported for Mean Timed Up and Go test in seconds and SD for standard deviation for each condition based on the original data without the outlier trials.
