# Supplementary material for: Benefits of dance for Parkinson’s: The music, the moves, and the company
Source: PLoS One. 2022 Nov 21;17(11):e0265921. doi: 10.1371/journal.pone.0265921 (PMC9678293; doi:10.1371/journal.pone.0265921)
Supplement: S1 File — (DOCX) [file pone.0265921.s005.docx]

**S5 Supplementary Information. Exploratory analysis with hours of dance class experience as an additional fixed effect.** According to the Akaike information criterion, the model with Music, Time, and Experience as fixed effects (M*T*E + (1|p)) showed a better prediction for our data than the model without Experience (-513.66 vs. -526.14), Chisq(4)=20.48, p < 0.001.

- This model showed a significant fixed effect of music, t(265)=-1.976, p < 0.049, ES = -0.035, SE = 0.018 but time as a fixed effect was not significant.
- Experience also showed as a significant main effect, improving performance, t(25.35)=-2.079, p < 0.047, ES = -0.001, SE = 0.001.
- Moreover, experience had a significant influence on time, t(265)=-2.839, p < 0.005, ES = -0.001 , SE = 0.0002.

In other words, the more hours participants danced with music, the stronger the interaction of music and movement on our observed effects, showing a lesser short-term but stronger long-term effect in line with the higher sensorimotor synchronisation ability previously reported. However, Shapiro-Wilk test normality was borderline (W= 0.991, p = 0.080).
