# Supplementary material for: Benefits of dance for Parkinson’s: The music, the moves, and the company
Source: PLoS One. 2022 Nov 21;17(11):e0265921. doi: 10.1371/journal.pone.0265921 (PMC9678293; doi:10.1371/journal.pone.0265921)
Supplement: Attachment — Submitted filename: Response.docx [file pone.0265921.s007.docx]

Response to reviewers

We were asked to #1 clarify the notion of our sample size, to #2 clarify the model and table values, to #3 provide a table with mean ±SD, and to #4 move the diagnostics plot into the supplement. We have addressed all points in the manuscript and clarify our changes below:

#1 We have now clarified the resource limitations by including the following two sentences in the participant section: “Due to resource limitations, it was not practical to set a sample size based on a priori power analysis. Our sample was therefore a convenience sample.”

And in the limitations, we rephrased our section on the participant sample size. Please note that we feel that including the phrase that “the study is underpowered” does not provide an accurate description of what power is or does. It now reads: “Another limitation of our study is that that we had resource limitations that restricted the number of participants. It can thus be argued that our design is underpowered – or in other words, less sensitive to our alternative hypotheses H1, H2, and H3. The risk for falsely rejecting small to medium effects is indeed relatively high with a small sample size. The risk for falsely rejecting small to medium effects is indeed relatively high with a small sample size. The results of our analyses should thus be interpreted with caution when even small effects are effects of interest as they are here;…… Clearly, our results would benefit from replication in form of a clinical trial.

To clarify our stance: we agree that in psychological research, power analyses are an important tool. It allows to determine the smallest sample size that is suitable to detect an effect of a particular size for a given test at the desired level of significance. Notably, our G*power analysis (described in our previous response) showed that for a small to medium effect, our study was not underpowered. In general, there is however an argument that post-hoc power analyses should not be conducted. Also note that dealing with power in linear mixed model analyses is different altogether. Firstly, it is not common to conduct a power analysis for mixed linear model analyses. They are so powerful because they deal better with variation in the data than standard repeated measures designs. Secondly, we have previously explained the specific issue of our relatively small sample size in that it increases the risk for falsely rejecting our H1.

We have now added information and changed this part (see above highlighted section) regarding issues of the small sample size and what that means for the interpretation of our data. We feel that our response provides the reader with more specific information regarding the challenges of a relatively small sample size which we feel addresses the PLOS One’s publication criteria better than a more general description of a study being underpowered.

#2 There seems to have been some confusion regarding the terminology of the “null-model” and the “alternative model”. We thank the reviewer for pointing this out and have now changed this. It reads: “When normality of the underlying residuals could be assumed, we compared the null-model with the alternative ‘full-model’, including the predictors conditions music and time as fixed effects and participants as a random nested effect (M*T + (1|p)).” We have also clarified all references to the two models throughout the manuscript.

As for the tables, please note that table 3 is the descriptive statistics that we were asked to include. The values describe the data and are not representing a model and table 4 shows contrasts and pairwise effects according to least-squares means which are also not representing a model. Therefore, it would be incorrect to state that “these values reflect a linear mixed model….” but because other readers may have the same uncertainty about the tables, we have added further information regarding for which model the values were obtained.

#3 We are not sure why the reviewer asks us for this additional data since all data from our analyses are available in sufficient detail in the manuscript and the tables as well as the raw data which is accessible through the OSF (Open Science Framework). We think it is important to highlight that mean and SD are not original outcome measure values of a linear mixed model analysis. Nevertheless, since other readers may have similar doubts, we have created a table with what we think the reviewer asked us for and included this in the supplement (S2).

#4 We agree that the regression diagnostics plots are better placed in the supplement and have moved them there.
